# From randomized controlled trial to real world clinical evidence: effectiveness and engagement of in-home virtual reality pain treatment for chronic pain in older adults

**DOI:** 10.3389/fpain.2025.1694007

**Published:** 2025-12-10

**Authors:** Todd Maddox, Josh Sackman, Emily Judge, Roselani Maddox, Robert Bonakdar, Beth D. Darnall

**Affiliations:** 1 AppliedVR, Inc., Van Nuys, CA, United States; 2 Scripps Center for Integrative Medicine, La Jolla, CA, United States; 3 Department of Anesthesiology, Perioperative and Pain Medicine, Stanford University School of Medicine, Palo Alto, CA, United States

**Keywords:** virtual reality, chronic lower back pain, immersive therapeutic, cognitive behavioral therapy (CBT), VR

## Abstract

Chronic lower back pain prevalence is greatest among older adults. Older adults (65+) often have multiple comorbidities and are more likely to have high-impact chronic pain that significantly impacts daily activities and function. Owing to greater pain burden, older adults are prime candidates for low-risk, in-home pain treatment. The goal of the present study was two-fold: (1) to examine clinical effectiveness of an FDA-Authorized Skills-Based Virtual Reality delivered therapy for chronic lower back pain in adults (18–64) and older adults (65+) by conducting a secondary analysis of a randomized controlled trial (*N* = 505), and (2) to examine engagement rates with the Skills-Based Virtual Reality delivered therapy in adults and older adults in the randomized controlled trial sample, and a separate real-world clinical sample (*N* = 2460). The clinical effectiveness analysis found that adults and older adults with chronic lower back pain showed statistically equivalent and clinically meaningful reductions in pain intensity and pain interference that were durable to 12-months post-treatment along with parallel improvements in sleep, depression, and physical disability. Adult and older adult high-impact chronic pain patients showed greater pain reductions than lower impact chronic pain patients with the majority shifting to lower impact chronic pain status at end-of-treatment that was maintained at 12-months post-treatment. VR program engagement analysis showed that older adults evidenced higher engagement in VR relative to adults in both the randomized controlled trial and real-world clinical sample. Together these results challenge common misperceptions about older adults and suggest that this Skills-Based VR therapy is an accessible solution for chronic lower back pain in older adults that leads to strong clinical outcomes and high VR program engagement.

**Clinical Trial Registration:**

ClinicalTrials.gov, identifier NCT05263037.

## Introduction

The prevalence of chronic lower back pain (CLBP) increases with age (1). Older adults with CLBP (65+ years of age) are more likely to have multiple comorbidities (2), high-impact chronic pain (3) and are less likely to receive non-pharmacologic care (4). Older adults are ideal candidates for low-risk and accessible in-home interventions, such as virtual reality–delivered therapy. Even so, important questions remain about treatment effectiveness and patient engagement with digital technologies in ages 65 and above (5–8). Recent studies show that the effectiveness of digital therapeutics in general varies across age, with older adults often experiencing less benefit (5–8). To reduce these disparities, researchers have highlighted the importance of ensuring broad accessibility (e.g., not requiring Wi-Fi for the digital therapy to be delivered) and increased ease-of-use (e.g., minimal time demands, at-home use, and intuitive navigation). It is important that new digital therapeutic technologies specifically evaluate these factors within the context of key demographic factors such as age, especially for conditions such as CLBP that increase in prevalence with age.

In a recent study, Maddox et al. (9) conducted a secondary analysis of a large-sample randomized controlled trial (RCT) (10) and found that pain intensity and pain interference reductions and VR program engagement at end-of-treatment for an FDA-authorized, in-home 56-session Skills-Based Virtual Reality Therapy for CLBP was generally unaffected by a number of sociodemographic factors including age (adults: 18–64 vs. older adults: 65+). One notable exception was that older adults were significantly more engaged with the program [where program engagement was defined as the number of VR sessions (out of a total of 56)] than adults, although both groups were highly engaged.

The primary aims (and sub-aims) of the present research are summarized in Table 1 along with details on the relevant study sample used to address each aim. Aim 1 was to examine age-effects on the clinical effectiveness of the Skills-Based Virtual Reality Therapy for CLBP. The relevant study sample used to address Aim 1 is the Maddox et al. RCT (10). Aim 1 consists of three sub-aims. Aim 1a examines age-effects (adults vs. older adults) on the pain intensity and pain interference reduction (primary endpoints) at end-of-treatment and 12-months post-treatment. Aim 1b examines age-effects (adults vs. older adults) on anxiety, sleep disturbance, depression, and physical disability reductions (secondary endpoints) at end-of-treatment and 12-months post-treatment. Research suggests that anxiety, sleep disturbance, depression, and physical disability are all more prevalent and often more severe in older adults with CLBP (11–13). It is important to determine if the Skills-Based Virtual Reality Therapy for CLBP reduces anxiety, sleep disturbance, depression and physical disability in older adults with CLBP. Aim 1c was exploratory and examined the relationship between age and pain impact group: high-impact chronic pain (BPI Interference ≥ 7) vs. lower impact chronic pain (BPI Interference < 7) (3, 14–16) on clinical effectiveness at end-of-treatment and 12-months post-treatment. Our previous research showed larger pain intensity and interference reductions for high-impact chronic pain than lower impact chronic pain following Skills-Based VR therapy (17). In addition, we found that 70% of high-impact chronic pain patients were reclassified as lower impact chronic pain at end-of-treatment with 67% remaining lower impact chronic pain at 12-months post-treatment. Given the heavy use of the healthcare system for high-impact chronic pain, the reclassification to lower impact chronic pain suggests a potential reduction in healthcare utilization and enhanced quality of life. We were interested in determining if this pattern was observed for adults and older adults at end-of-treatment and 12-months post-treatment. This is especially important given the fact that the prevalence of high-impact chronic pain increases with age (18).

**Table 1:** Organization of the study analysis including study aims and sub-aims along with the relevant study sample used to test each Aim.

Study aims		Relevant study sample	
Primary Aims	Sub-Aims	Maddox et al. Randomized Controlled Trial (RCT)	Real-World Clinical Sample
Aim 1: Examine Age-Effects on Clinical Effectiveness	Aim 1a: Age-effects on pain intensity and pain interference reduction (primary endpoints) at end-of-treatment and 12-months post-treatment	✓	
	Aim 1b: Age-effects on anxiety, sleep disturbance, depression and physical disability reduction (secondary endpoints) at end-of-treatment and 12-months post-treatment	✓	
	Aim 1c (Exploratory): Age and pain impact group effects on primary and secondary endpoints at end-of-treatment and 12-months post-treatment	✓	
Aim 2: Examine Age-Effects on VR Program Engagement		✓	✓

Aim 2 examines age-effects on the engagement with the Skills-Based Virtual Reality Therapy for CLBP. The relevant study samples used to address Aim 2 are the Maddox et al. RCT (10) and a real-world clinical sample of 2,460 patients who have received a prescription for the FDA-authorized Skills-Based Virtual Reality Therapy from a healthcare professional as of October 2025. This real-world clinical sample (independent of the RCT) provides critical insights into engagement with the VR-delivered therapy when it is clinically prescribed.

Given our previous findings (9), we predict that adults and older adults will show clinically meaningful and statistically equivalent levels of pain reduction at end-of-treatment and at 12-months post-treatment (Aim 1a). We expect statistical equivalence across adults and older adults in the secondary endpoint reductions as well at end-of-treatment and 12-months post-treatment (Aim 1b). We also expect the high-impact chronic pain advantage observed in the full RCT data set (17) to hold for adults and older adults at both time points (Aim 1c). Finally, we predict that the older adult VR engagement advantage observed in the Maddox et al. RCT sample will replicate in the real-world clinical sample (Aim 2).

## Methods

### Study design, participants and randomization

The Methods, CONSORT diagram and analyses at end-of-treatment and 12-month post-treatment for the primary decentralized randomized controlled trial are published (10, 19). Although the secondary analysis presented in this report focuses exclusively on the Skills-Based VR therapy, for completeness, we briefly describe some of the trial details, including the Sham VR group. A national sample of *N* = 1,093 individuals with self-reported non-malignant lower back pain ≥ 3 months duration and with average pain intensity and pain interference (20) ≥4/10 in the past month were enrolled and randomized 1:1 to one of two 56-session VR-delivered programs: (1) Skills-Based VR (immersive pain relief skills); or (2) Sham VR [two-dimensional (2D) nature content]. The Skills-Based VR program (RelieVRx^©^) is an FDA *de novo* authorized immersive, multi-modal VR treatment program for cLBP. The 56-session program integrates evidence-based skills such as diaphragmatic breathing, biofeedback, cognition and emotion regulation, mindfulness, and pain education into a 56-session therapeutic journey. Daily immersive experiences are organized into 8 7-session themes. Interactive biodata-enabled therapeutics that capture user exhalation through an embedded microphone provide synchronized 3D visual and auditory biofeedback. The duration of daily VR treatment sessions ranges from 2 to 16 min, and the patient is urged to complete one session per day. The VR device is shipped to the patient's home along with instructions for use. Patients are also provided with contact information for customer support should it be needed.

The resulting sample was demographically diverse (female: 77%; non-Caucasian: 32%; high school or less education: 19%; mean age: 50.8) and clinically severe (baseline pain intensity = 6.6; baseline pain interference = 6.2, disability = 42%; severe/completely disabled). Adults (18–64) had a mean age of 46.5 (SD = 10.9) and older adults (65+) a mean age of 70.5 (SD = 4.8). The fixed-sequence Skills-Based VR-delivered program is an immersive multimodal pain self-management program that incorporates evidence-based principles of CBT, mindfulness, and pain neuroscience education. Consistent with VR-CORE trial guidelines, an active control group using non-immersive 2D content within a VR headset was selected as the most rigorous VR placebo (21). Sham VR was designed to mimic the VR device experience without 3D immersion, verbal and pain relief skills content. Sham VR involved a fixed sequence of 56 2D nature videos overlaid with neutral music. The study protocol was approved by the WCG Institutional Review Board (Puyallup, WA) in December 2021, and followed the Consolidated Standards of Reporting Trials (CONSORT) reporting guidelines. Study data were collected from January 31, 2022 to October 31, 2024.

### Self-report measures

#### Demographics

Age, gender, race, ethnicity, height, weight, annual household income, and pain comorbidities including aarachnoiditis, CRPS, degenerative disc disease, epidural fibrosis, failed back surgery syndrome, fibromyalgia, lumbar disc herniation, lupus. lyme disease, osteoporosis, pain associated with multiple sclerosis, peripheral neuropathy, phantom limb pain, osteoarthritis, rheumatoid arthritis, scoliosis, spinal stenosis, and spondylolisthesis.

#### Brief pain inventory (BPI): pain intensity

The BPI Pain Intensity uses a single item to measure pain intensity over the last 24 h using a 0–10 numeric rating scale (20). This measure was administered at baseline, end-of-treatment, and 12-months post-treatment. Five additional administrations of the BPI pain intensity were included (one per day) during the 5 days following initial baseline administration, with a requirement that at least 2 be completed. These were averaged with the baseline BPI to obtain the overall baseline BPI intensity.

#### Brief pain inventory (BPI): pain interference

The BPI Pain Interference measures how much pain interferes with seven domains of daily life (enjoyment of life, general activity, mood, normal work, relations with other people, sleep, and walking ability) over the last 24 h using a 0–10 numeric rating scale (20). The scores on the seven items are averaged to generate a global pain interference score. This measure was administered at baseline, end-of-treatment, and 12-month post-treatment. Five additional administrations of the BPI pain interference were included (one per day) during the 5 days following initial baseline administration, with a requirement that at least 2 be completed. These were averaged with the baseline BPI to obtain the overall baseline BPI interference scores.

#### PROMIS anxiety (version 7a)

The PROMIS Anxiety is a 7-item survey that assesses an individual's perception of their anxiety level over the past 7 days (22). Raw scores on the PROMIS Anxiety survey are converted to T-scores, which range from 36.3–82.7. This measure was administered at baseline, end-of-treatment, and 12-month post-treatment.

#### PROMIS sleep disturbance (version 8b)

The PROMIS Sleep Disturbance is an 8-item survey that assesses an individual's perception of sleep quality, sleep depth, and restoration associated with sleep over the past 7 days (23). Raw scores on the PROMIS Sleep Disturbance survey are converted to T-scores, which range from 28.9–76.5. This measure was administered at baseline, end-of-treatment, and 12-month post-treatment.

#### PROMIS depression (version 8b)

The PROMIS Depression is an 8-item survey that assesses the frequency and severity of depressive symptoms over the past 7 days (22). It covers topics such as feelings of worthlessness, hopelessness, and loss of interest in activities. Raw scores on the PROMIS Depression survey are converted to T-scores, which range from 37.1–81.1. This measure was administered at baseline, end-of-treatment, and 12-months post-treatment.

#### Oswestry disability Index (ODI) (version 2.1b)

The ODI is a 10-item survey that assesses how lower back pain affects one's ability to manage in everyday life (24). Scores range from 0 to 100 and reflect the level of disability. This measure was administered at baseline, end-of-treatment, and 12-months post-treatment.

These are all widely utilized and well validated measures that are effective for measuring changes in chronic lower back pain and relevant comorbidities following VR-delivered therapy.

### Objective measure

#### VR program engagement

VR program engagement was objectively assessed as the number of sessions initiated with at least 45% of the session completed. Although somewhat arbitrary, previous user testing showed that patients either completed well below 45% of a VR experience (essentially engaging briefly then skipping the experience) or completed approximately 40% or more. Because our target was at least 50% completed, we designated the threshold as the midpoint at 45%. The number of sessions completed by each participant was captured from the VR device after treatment and upon device return.

### Statistical analysis

Aim 1: Aim 1 focused exclusively on analysis of the Maddox et al. RCT sample. Continuous and categorical baseline variables were summarized using mean (SD) and count (percentage), respectively. The standardized mean differences between older adult (65+) and adult (18–64) groups were calculated. Between-group comparisons were conducted using t-tests (for continuous baseline variables) or Fisher exact tests (for categorical baseline variables). The modified intention-to-treat analysis set (i.e., all participants randomized who received a VR device) was used to investigate Skills-Based VR therapy effects in 65+ vs. 18–64 adults. To evaluate Skills-Based VR therapy effects in 65+ vs. 18–64 adults on the two primary endpoints (Aim 1a; BPI pain interference and BPI pain intensity), separate mixed models for repeated measure [MMRM (25, 26)]; analysis was conducted with the dependent variable being the relevant endpoint score (BPI interference or BPI intensity) and independent variables being group (65+ vs. 18–64 adult) and time (i.e., baseline, end-of-treatment or 12-months post-treatment). Group effects were summarized as the estimated between-group difference in endpoint score reductions from baseline to end-of-treatment or baseline to 12-months post-treatment and the associated 95% CI. Because the MMRM analysis included baseline endpoint scores in repeated measurements, the analysis adjusted for potential imbalance in baseline endpoint score by treating the baseline value as a covariate. We also include race/ethnicity, number of comorbidities and BMI as covariates since these differed across age groups (see Table 2 to be discussed shortly). Similar analyses examined the group effect for the four secondary endpoints (Aim 1b; PROMIS Anxiety, PROMIS Sleep Disturbance, PROMIS Depression and ODI). For the primary endpoints, the Bonferroni adjusted statistical significance level for comparing groups was set at a 2-sided level of.025 to adjust for 2 comparisons. For the secondary endpoints, the Bonferroni adjusted statistical significance level for comparing groups was set at a 2-sided level of.0125 to adjust for 4 comparisons.

**Table 2:** Participant demographic characteristics and select baseline measures in the mITT analysis Set.

Measure	RelieVRx: 18–64 (*n* = 449)	RelieVRx: 65+ (*n* = 87)	*P*
Age	46.5 (10.9)	70.5 (4.8)	<0.0001
Gender			0.25
Female	348 (77.5)	63 (72.4)	
Male	100 (22.3)	24 (27.6)	
Non-binary	1 (0.2)	0 (0.0)	
Race and Ethnicity			0.025
American Indian/Alaska Native	4 (0.9)	0 (0.0)	
Asian/Pacific Islander	11 (2.4)	1 (1.1)	
Black/African American	75 (16.7)	6 (6.9)	
Caucasian	296 (65.9)	74 (85.1)	
Hispanic/Latin	16 (3.6)	2 (2.3)	
Multiracial	47 (10.5)	4 (4.6)	
Household Annual Income			0.35
<$60,000	267 (59.5)	47 (54.0)	
≥$60,000	182 (40.5)	40 (46.0)	
Number of Pain Comorbidities			<0.0001
0	200 (44.5)	15 (17.2)	
≥1	228 (50.8)	64 (73.6)	
Non reported	21 (4.7)	8 (9.2)	
Body mass index	32.1 (13.1)	29.4 (7.1)	0.007
BPI Pain Intensity	6.7 (1.5)	6.2 (1.3)	<0.001
BPI Pain Interference	6.3 (1.8)	5.6 (1.7)	<0.001
PROMIS Anxiety	56.6 (9.2)	53.5 (8.3)	0.002
PROMIS Sleep Disturbance	61.3 (7.0)	57.5 (7.2)	<0.0001
PROMIS Depression	55.2 (9.3)	53.6 (9.4)	0.14
Oswestry Disability Index (ODI)	41.6 (15.8)	40.6 (14.2)	0.55

Data are presented as *n* (%) or mean (SD). *P*-value from t-test or Fisher's exact test comparing 18–64 to 65+ Adults.

For the exploratory pain impact group analysis (Aim 1c), we classified 65+ and 18–64 adults as high-impact chronic pain or lower impact chronic pain using a baseline BPI Pain Interference threshold of 7 (27). Since the goal was to determine if larger pain reductions were observed for high-impact chronic pain than lower impact chronic pain patients for each age groups, MMRM analyses were conducted separately for each age group with the dependent variable being the relevant endpoint score and the independent variables being pain impact group (high-impact chronic pain vs. lower impact chronic pain) and time (i.e., baseline, end-of-treatment or 12-months post-treatment).

Aim 2: Aim 2 focused on analysis of the Maddox et al. RCT sample and the real-world clinical sample. For both samples, we began by calculating the number of VR experiences completed by each participant (in the RCT) and patient (in the real-world clinical sample) with the restriction that we included only individuals who completed at least one VR-delivered experience. For each sample and separated by age group (adults vs. older adults) we computed the sample size (N) and average number of sessions completed (out of a total of 56). We also computed the “engagement” rate, defined as the percentage of patients who completed at least 24 sessions. The 24-session threshold in the engagement rate was derived from our pivotal RCT (28) where we showed that 24 sessions represented a minimum dose needed to obtain clinically meaningful pain reductions at end-of-treatment. Finally, we computed the “adherence: rate, defined as the percentage of patients who completed at least 40 sessions”. The 40-session threshold in the adherence rate is not defined empirically from an RCT but rather represents a target goal where patients complete at least 40 sessions (on average 5 sessions/week across the 8-week therapeutic program).

## Results

### Participants

Table 2 displays the baseline demographic and clinical characteristics by age group (18–64 vs. 65+ adults) for the Skills-Based VR therapy participants. Older adults (*N* = 87) represent 16% of the total sample. The 65+ group reported less racial diversity being predominantly Caucasian (*p* = 0.025), a larger number of pain comorbidities (*p* < 0.0001), and lower BMI (*p* = 0.007) than the 18–64 adult group. For baseline clinical symptoms, the older adult group reported significantly lower levels of pain intensity, pain interference, anxiety and sleep disturbance with *p*-value group differences ranging from 0.007 to <0.0001. As outlined in the Methods section, the use of MMRM and the addition of several covariates helps account for these group differences in the statistical analysis.

### Aim 1a: age-effects on pain intensity and pain interference reductions (primary endpoints) at end-of-treatment and 12-months post-treatment

MMRM yielded a non-significant age group x time interaction for BPI Pain Intensity (*p* = 0.96). Although the magnitude of the pain intensity reductions did not differ across age groups, the pain intensity reductions from baseline to end-of-treatment for both groups were statistically significant [18–64 adults: 1.91 (95% CI, 1.72–2.10), *p* < 0.001; 65+ adults: 1.93 (95% CI, 1.50–2.37), *p* < 0.001], and these were sustained to 12-months post-treatment [18–64 adults: 1.71 (95% CI, 1.48–1.93), *p* < 0.001; 65+ adults: 1.52 (95% CI, 1.06–1.99), *p* < 0.001]. [See Table 3 for details]. MMRM yielded a non-significant age group x time interaction for BPI Pain Interference (*p* = 0.62). Despite the lack of an age group difference, the pain interference reductions from baseline to end-of-treatment for both groups were statistically significant [18–64 adults: 2.21 (95% CI, 2.01–2.41), *p* < 0.001; 65+ adults: 2.15 (95% CI, 1.77–2.52), *p* < 0.001], and these were sustained to 12-months post-treatment [18–64 adults: 1.98 (95% CI, 1.74–2.22), *p* < 0.001; 65+ adults: 1.65 (95% CI, 1.14–2.16), *p* < 0.001].

**Table 3:** RelieVRx between-group differences at end-of-treatment and 12-months post-treatment for the primary and secondary endpoints.

	Mean (SD) score [No of participants]		RelieVRx: 18–64 Minus 65+ Reduction from Baseline^a^	
Time	RelieVRx: 18–64	RelieVRx: 65+	Estimate (SE) [95% CI]	*P* value^b^
Brief Pain Inventory (BPI) Pain Intensity^c^				
Baseline	6.72 (1.49) [449]	6.16 (1.33) [87]		
End-of-Treatment	4.78 (2.20) [400]	4.16 (1.92) [79]	−0.02 (0.24) [−0.49–0.45]	0.92
12-Months Post-Treatment	5.04 (2.32) [357]	4.56 (2.05) [72]	0.18 (0.28) [−0.36–0.72]	0.51
Brief Pain Inventory (BPI) Pain Interference^d^				
Baseline	6.34 (1.84) [449]	5.58 (1.65) [87]		
End-of-Treatment	4.07 (2.02) [400]	3.36 (1.97) [79]	0.06 (0.24) [−0.42–0.54]	0.81
12-Months Post-Treatment	4.37 (2.58) [357]	3.85 (2.43) [72]	0.33 (0.30) [−0.26–0.91]	0.27
PROMIS Anxiety^e^				
Baseline	56.61 (9.21) [449]	53.50 (8.35) [87]		
End-of-Treatment	56.24 (9.29) [400]	51.06 (8.02) [79]	−1.77 (0.97) [−3.67–0.13]	0.07
12-Months Post-Treatment	55.79 (9.87) [357]	50.77 (7.95) [72]	−1.12 (1.14) [−3.36–1.13]	0.33
PROMIS Sleep Disturbance^f^				
Baseline	61.35 (6.96) [449]	57.50 (7.21) [87]		
End-of-Treatment	56.85 (8.05) [400]	53.40 (8.25) [79]	0.66 (0.90) [−1.10–2.42]	0.46
12-Months Post-Treatment	55.94 (8.36) [357]	52.68 (7.53) [72]	1.44 (1.05) [−0.63–3.50]	0.17
PROMIS Depression^g^				
Baseline	55.21 (9.26) [449]	53.59 (9.35) [87]		
End-of-Treatment	54.14 (9.71) [400]	52.05 (8.60) [79]	−0.50 (0.97) [−2.40–1.39]	0.60
12-Months Post-Treatment	53.72 (10.44) [357]	50.75 (9.25) [72]	−1.13 (1.23) [−3.54–1.29]	0.36
Oswestry Disability Index^h^				
Baseline	41.62 (15.80) [449]	40.60 (14.24) [87]		
End-of-Treatment	32.86 (17.05) [400]	34.68 (13.97) [79]	3.68 (1.55) [0.63–6.74]	0.018
12-Months Post-Treatment	30.95 (19.84) [357]	32.76 (15.73) [72]	3.88 (2.00) [−0.06–7.81]	0.05

a Reduction from Baseline to End-of-Treatment (or 12-Months Post-Treatment).

b Bonferroni corrected.

c Score range from 0 to 10 with higher scores indicating greater pain intensity.

d Score range from 0 to 10 with higher scores indicating greater pain interference.

e Score range from 36.3–82.7 with higher scores indicating greater anxiety.

f Score range from 28.9–76.5 with higher scores indicating greater sleep disturbance.

g Score range from 37.1–81.1 with higher scores indicating greater depression.

h Score range from 0 to 100 with higher scores indicating greater disability.

### Aim 1b: age-effects on anxiety, sleep disturbance, depression and physical disability reductions (secondary endpoints) at end-of-treatment and 12-months post-treatment

MMRM yielded a non-significant age group x time interaction for PROMIS Anxiety (*p* = 0.26). Although the magnitude of the anxiety reductions did not differ across age groups, the anxiety reductions from baseline to end-of-treatment and baseline to 12-months post-treatment were statistically significant for 65+ adults [baseline to end-of-treatment: 2.00 (95% CI, 0.65–3.35), *p* = 0.004; baseline to 12-months post-treatment: 1.82 (95% CI, 0.24–3.39), *p* = 0.0120] but not 18–64 adults [baseline to end-of-treatment: 0.23 (95% CI, 0.57–1.04), *p* = 0.57; baseline to 12-months post-treatment: 0.70 (95% CI, 0.26–1.66), *p* = 0.15]. MMRM yielded a non-significant age group x time interaction for PROMIS Sleep Disturbance (*p* = 0.58). Despite the lack of age group differences, the sleep disturbance reductions from baseline to end-of-treatment for both groups were statistically significant [18–64 adults: 4.50 (95% CI, 3.76–5.25), *p* < 0.001; 65+ adults: 3.84 (95% CI, 2.56–5.12), *p* < 0.001], and these were sustained to 12-months post-treatment [18–64 adults: 5.29 (95% CI, 4.43–6.15), *p* < 0.001; 65+ adults: 3.86 (95% CI, 2.13–5.58), *p* < 0.001]. MMRM yielded a non-significant age group x time interaction for PROMIS Depression (*p* = 0.62). Although the magnitude of the depression reductions did not differ across age groups, the depression reductions from baseline to end-of-treatment was statistically significant for 18–64 adults [0.92 (95% CI, 0.13–1.72), *p* = 0.0120], and was trending (but did not meet statistical significance after correction for multiple comparisons) in 65+ adults [1.43 (95% CI, 0.05–2.81), *p* = 0.021]. Interestingly, the depression reductions from baseline to 12-months post-treatment were significant for both age groups [18–64 adults: 1.45 (95% CI, 0.42–2.48), *p* = 0.003; 65+ adults: 2.57 (95% CI, 0.89–4.26), *p* = 0.003]. MMRM yielded a non-significant (after correction for multiple comparisons) age group x time interaction for the Oswestry Disability Index (*p* = 0.04). Even so, while the disability reductions from baseline to end-of-treatment for both groups were statistically significant [18–64 adults: 8.54 (95% CI, 7.25–9.82), *p* < 0.001; 65+ adults: 4.85 (95% CI, 2.62–7.08), *p* < 0.001], and these were sustained to 12-months post-treatment significant [18–64 adults: 10.49 (95% CI, 8.82–12.15), *p* < 0.001; 65+ adults: 6.61 (95% CI, 3.65–9.57), *p* < 0.001] they were considerably larger for 18–64 than 65+ adults.

### Aim 1c: pain impact group differences for 65± and 18–64 adults at end-of-treatment and 12-months post-treatment

#### 65± adults, primary endpoints

MMRM yielded a non-significant pain impact group x time interaction for BPI Pain Intensity (*p* = 0.41). Even so, the magnitude of the pain intensity reductions were larger for the high-impact chronic pain (end-of-treatment = 2.50; 12-months post-treatment = 1.82) than the lower impact chronic pain (end-of-treatment = 1.79; 12-months post-treatment = 1.45) group at both time points (see Table 4), and the pain reductions were statistically significant for both groups at both time points (largest *p*-value = 0.001). MMRM yielded a non-significant pain impact group x time interaction for BPI Pain Interference (*p* = 0.20). Even so, the magnitude of the pain interference reductions were larger for the high-impact chronic pain (end-of-treatment = 2.72; 12-months post-treatment = 2.27) than the lower impact chronic pain (end-of-treatment = 2.00; 12-months post-treatment = 1.49) group at both time points (see Table 4), and the pain reductions were statistically significant for both groups at both time points (largest *p*-value = 0.001).

**Table 4:** RelieVRx pain impact group differences from baseline to end-of-treatment and baseline to 12-months post-treatment for the primary and secondary endpoints by Age group.

	RelieVRx: 18–64		RelieVRx: 65+	
	Mean (SD) score [No of participants]		Mean (SD) score [No of participants]	
Time	Lower impact chronic pain	High-impact chronic pain	Lower impact chronic pain	High-impact chronic pain
Brief pain inventory (BPI) pain intensity^a^				
Baseline Minus End-of-Treatment	1.76** (1.79) [253]	2.17** (2.19) [147]	1.79** (1.83) [63]	2.50** (2.33) [16]
Baseline Minus 12-Months Post-Treatment	1.45** (2.09) [217]	2.11** (2.22) [140]	1.45** (1.85) [57]	1.82* (2.40) [15]
Brief pain inventory (BPI) pain interference^b^				
Baseline Minus End-of-Treatment	1.94** (1.72) [253]	2.67** (2.40) [147]	2.00** (1.47) [63]	2.72** (2.30) [16]
Baseline Minus 12-Months Post-Treatment	1.57** (2.16) [217]	2.61** (2.45) [140]	1.49** (1.97) [57]	2.27** (2.78) [15]
PROMIS anxiety^c^				
Baseline Minus End-of-Treatment	0.24 (7.14) [253]	0.23 (9.72) [147]	2.01** (5.99) [63]	1.96 (6.36) [16]
Baseline Minus 12-Months Post-Treatment	1.42** (7.83) [217]	0.43 (10.94) [140]	1.75* (6.48) [57]	2.07 (7.67) [15]
PROMIS sleep disturbance^d^				
Baseline Minus End-of-Treatment	3.43** (6.86) [253]	6.36** (8.32) [147]	3.84** (5.46) [63]	3.87* (6.80) [16]
Baseline Minus 12-Months Post-Treatment	4.25** (7.55) [217]	6.90** (9.11) [140]	3.49** (6.60) [57]	5.23 (9.77) [15]
PROMIS depression^e^				
Baseline Minus End-of-Treatment	1.27** (6.98) [253]	0.33 (9.80) [147]	1.24 (5.23) [63]	2.15 (9.16) [16]
Baseline Minus 12-Months Post-Treatment	2.00** (7.81) [217]	0.60 (12.46) [140]	1.57 (6.47 [57]	6.37* (8.58) [15]
Oswestry disability index^f^				
Baseline Minus End-of-Treatment	7.11** (11.89) [253]	10.99** (14.59) [147]	4.48** (9.55) [63]	6.32* (11.67) [16]
Baseline Minus 12-Months Post-Treatment	9.58** (14.80) [217]	11.90** (17.72) [140]	4.62** (10.44) [57]	14.18** (17.10) [15]

a Score range from 0 to 10 with higher scores indicating greater pain intensity.

b Score range from 0 to 10 with higher scores indicating greater pain interference.

c Score range from 36.3–82.7 with higher scores indicating greater anxiety.

d Score range from 28.9–76.5 with higher scores indicating greater sleep disturbance.

e Score range from 37.1–81.1 with higher scores indicating greater depression.

f Score range from 0 to 100 with higher scores indicating greater disability.

* *p* < 0.05.

** *p* < 0.01 for pain reduction from baseline to end-of-treatment or baseline to 12-months post-treatment.

Although the HICP 65+ adult sample is small, we computed the number and percentage of high-impact chronic pain 65+ adults reclassified as lower impact chronic pain at each time point. We found that 13 of the 16 high-impact chronic pain 65+ adults were reclassified as lower impact chronic pain at end-of-treatment (81%) with 8 of 15 high-impact chronic pain 65+ adults remaining classified as lower impact chronic pain at 12-months post-treatment (53%).

#### 65± adults, secondary endpoints

MMRM yielded a non-significant pain impact group x time interaction for PROMIS Anxiety (*p* = 0.95). At end-of-treatment the anxiety reduction was slightly smaller for the high-impact chronic pain than the lower impact chronic pain group, and at 12-months post-treatment the anxiety reduction was slightly larger for the high-impact chronic pain than the lower impact chronic pain group (see Table 4). The anxiety reductions were statistically significant for the lower impact chronic pain group at both time points (largest *p*-value = 0.05) but were not statistically significant for the high-impact chronic pain group at either time point (smallest *p*-value = 0.24). MMRM yielded a non-significant pain impact group x time interaction for PROMIS Sleep Disturbance (*p* = 0.07). The magnitude of the sleep disturbance reductions were larger for the high-impact chronic pain than the lower impact chronic pain group at both time points (see Table 4), and the sleep disturbance reductions were statistically significant for both groups at both time points (largest *p*-value = 0.05). MMRM yielded a significant pain impact group x time interaction for PROMIS Depression (*p* = 0.02) that was characterized by a non-significant interaction at end-of-treatment (*p* = 0.60) but a significant interaction at 12-months post-treatment (*p* = 0.20). The magnitude of the depression reductions were larger for the high-impact chronic pain than the lower impact chronic pain group at both time points (see Table 4), but the depression reduction was only statistically significant for the high-impact chronic pain group at 12-months post-treatment (*p* = 0.01). MMRM yielded a significant pain impact group x time interaction for the Oswestry Disability Index (*p* = 0.02) that was characterized by a non-significant interaction at end-of-treatment (*p* = 0.51) but a significant interaction at 12-months post-treatment (*p* = 0.01). The magnitude of the disability reductions were larger for the high-impact chronic pain than the lower impact chronic pain group at both time points (see Table 4), and the disability reductions were statistically significant for both groups at both time points (largest *p*-value = 0.04).

#### 18–64 adults, primary endpoints

MMRM yielded a significant pain impact group x time interaction for BPI Pain Intensity (*p* < 0.001). This pattern was replicated at end-of-treatment relative to baseline (*p* = 0.04) and at 12-months post-treatment relative to baseline (*p* = 0.01). The magnitude of the pain intensity reductions were larger for the high-impact chronic pain (end-of-treatment = 2.17; 12-months post-treatment = 2.11) than the lower impact chronic pain (end-of-treatment = 1.76; 12-months post-treatment = 1.45) group at both time points (see Table 4), and the pain reductions were statistically significant for both groups at both time points (largest *p*-value = 0.001). MMRM yielded a significant pain impact group x time interaction for BPI Pain Interference (*p* < 0.001). This pattern was replicated at end-of-treatment relative to baseline (*p* < 0.001) and at 12-months post-treatment relative to baseline (*p* < 0.001). The magnitude of the pain interference reductions were larger for the high-impact chronic pain (end-of-treatment = 2.67; 12-months post-treatment = 2.61) than the lower impact chronic pain (end-of-treatment = 1.94; 12-months post-treatment = 1.57) group at both time points (see Table 4), and the pain reductions were statistically significant for both groups at both time points (largest *p*-value = 0.001).

We also computed the number and percentage of high-impact chronic pain 18–64 adults reclassified as lower impact chronic pain at each time point. We found that 100 of the 147 high-impact chronic pain 18–64 adults were reclassified as lower impact chronic pain at end-of-treatment (68%) with 96 of 140 high-impact chronic pain 18–64 adults remaining classified as lower impact chronic pain at 12-months post-treatment (69%).

#### 18–64 adults, secondary endpoints

MMRM yielded a non-significant pain impact group x time interaction for PROMIS Anxiety (*p* = 0.31). At end-of-treatment the anxiety reduction was slightly smaller for the high-impact chronic pain than the lower impact chronic pain group, and this difference was magnified at 12-months post-treatment (see Table 4). The anxiety reduction was statistically significant only for the lower impact chronic pain group at 12-months post-treatment (*p* = 0.008). MMRM yielded a significant pain impact group x time interaction for PROMIS Sleep Disturbance (*p* < 0.001). This pattern was replicated at end-of-treatment relative to baseline (*p* < 0.001) and at 12-months post-treatment relative to baseline (*p* = 0.003). The magnitude of the sleep disturbance reductions were larger for the high-impact chronic pain than the lower impact chronic pain group at both time points (see Table 4), and the sleep disturbance reductions were statistically significant for both groups at both time points (all *p*-value < 0.001). MMRM yielded a non-significant pain impact group x time interaction for PROMIS Depression (*p* = 0.47). The magnitude of the depression reductions were smaller for the high-impact chronic pain than the lower impact chronic pain group at both time points (see Table 4). The depression reductions were statistically significant for the lower impact chronic pain group at both time points (largest *p*-value = 0.004). MMRM yielded a significant pain impact group x time interaction for the Oswestry Disability Index (*p* = 0.02). This pattern was replicated at end-of-treatment relative to baseline (*p* = 0.004) but not at 12-months post-treatment relative to baseline (*p* = 0.18). The magnitude of the disability reductions were larger for the high-impact chronic pain than the lower impact chronic pain group at both time points (see Table 4), and the disability reductions were statistically significant for both groups at both time points (all *p*-value < 0.001).

### Aim 2: age-effects on skills-based VR therapy (RelieVRx) engagement in the maddox et al. RCT and a real-world clinical sample

The Skills-Based VR-Delivered therapy received *de novo* FDA authorization as a class II medical device in November 2021. The therapy was branded as RelieVRx^©^ and was launched commercially in November 2023. [For those interested in the development of the therapy, FDA authorization and commercialization see (29),]. As of October 2025, more than 2,400 patients have completed the prescription therapy, with hundreds more currently in therapy or awaiting shipment of the therapeutic device. These 2,400+ patients provide a real-world clinical sample of patients whose VR program engagement is worth exploring. Following completion of the therapy, the patient sends the device back to the AppliedVR warehouse and the VR program engagement data is downloaded from each device in a HIPAA compliant manner. We extracted only the engagement data (de-identified) and examined age effects on VR program engagement in this real-world clinical sample and compared patient engagement with those observed in the randomized clinical trial described above.

Table 5 summarizes patient/participant VR engagement across the clinical sample and RCT samples by adult status (65+ and 18–64 adults). The table includes the sample size (N), average number of sessions completed (out of a total of 56), the engagement rate, defined as the percentage of patients who completed at least 24 sessions, and the (28) adherence rate, defined as the percentage of patients who completed at least 40 sessions. The results were clear. First, the average number of sessions completed was high in the full sample (35.7) with more sessions being completed by older adults (40.7) than adults (34.3). Second, the engagement and adherence rates were high as well, also showing an older adult advantage. Specifically, 66% of the full sample were engaged (completing 24 or more sessions), with 75% of older adults and 63% of younger adults being engaged. Analogously, 55% of the full sample were adherent (completing 40 or more sessions), with 67% of older adults and 50% of younger adults meeting the more stringent adherence rate.

**Table 5:** VR program engagement in a real-world clinical sample of 2,400+ prescribed patients vs. a Large Sample RCT by Age Group.

Measure	Real-World Clinic Patients			RCT Participants		
	All	18–64	65+	All	18–64	65+
N	2,460	642	284	505	424	81
Ave Sessions Completed	35.7	34.3	40.7	37.5	35.8	46.4
% Engaged (24+)	66	63	75	75	72	94
% Adherent (40+)	55	50	67	60	55	85

Real-World Clinical Sample: Prescriptions as of October 2025. The total number of 18–64 and 65+ patients does not sum to the total for “All” because many patients did not provide a birthdate. An “engaged” participant or patient is defined as one who completes 24 or more sessions of the 56-session therapy. An “adherent” participant or patient is defined as one who completes 40 or more sessions of the 56-session therapy.

## Discussion

The prevalence of CLBP is high in the general population and increases with age (1). Older adults with CLBP are more likely to have multiple comorbidities (2), high-impact chronic pain (3) and are less likely to receive non-pharmacologic care (4). Thus, older adults should benefit from low-risk and accessible in-home interventions, such as virtual reality–delivered therapy. Even so, some studies find heterogeneity in the effectiveness of digital therapeutics as a function of age and often attribute this to the importance of ensuring broad accessibility (e.g., not requiring Wi-Fi for the digital therapy to be delivered) and increased ease-of-use (e.g., minimal time demands, at-home use, and intuitive navigation) (5, 7). We recently conducted a secondary analysis of a large sample RCT (10) and found that pain intensity and pain interference reductions, VR program engagement and device usability at end-of-treatment for a Skills-Based VR therapy (RelieVRx) was unaffected by age; in fact, older adults showed greater VR program engagement than adults (9).

The goals of the current study were (1) to explore age-effects on clinical effectiveness of a Skills-Based VR therapy (RelieVRx) at end-of-treatment and 12-months post-treatment, and (2) to test whether older adults sufficiently engage in therapeutic VR. To determine whether the treatment is effective in older adults, we conducted three secondary analyses of the Maddox et al. RCT (see Table 1 for the organization of the study analysis). First, we examined age-effects on the primary endpoints of pain intensity and pain interference at end-of-treatment and at 12-months post-treatment (Aim 1a). Second, we examined age-effects on the secondary endpoints of anxiety, sleep disturbance, depression, and physical disability at end-of-treatment and at 12-months post-treatment (Aim 1b). This is an important line of investigation because anxiety, depression, sleep disturbance and physical disability are all more prevalent and often more severe in older adults with CLBP (11–13) Third, we conducted an exploratory analysis examining the interaction between age and high-impact chronic pain vs. lower impact chronic pain on primary and secondary endpoint clinical effectiveness at end-of-treatment and 12-months post-treatment (Aim 1c). Our previous research showed larger pain intensity and interference reductions for high-impact chronic pain than lower impact chronic pain following Skills-Based VR therapy (17).

To test whether older adults sufficiently engage in the VR program (Aim 2), we examined the effect of age on VR program engagement in a real-world clinical sample of 2,460 patients who have received and completed the Skills-Based Virtual Reality Therapy, and compared that with VR program engagement data from our RCT. Examining VR program engagement across clinical and experimental samples is critical given the fact that clinical evidence consistently demonstrates that engagement with a digital therapy is a key determinant of its clinical effectiveness (30–32). Evaluating VR program engagement in older adults is especially important given recent studies showing that the effectiveness of digital therapeutics varies across age (5, 6).

The Aim 1 secondary analysis of the RCT yielded several interesting findings. First, clinically meaningful pain intensity and pain interference reductions were observed for adults and older adults at end-of-treatment that remained invariant across age at 12-months post-treatment. Second, statistically significant reductions in sleep disturbance and physical disability were observed at end-of-treatment and 12-months post-treatment for adults and older adults that were invariant across age. Statistically significant reductions in depression were observed at end-of-treatment and 12-months post-treatment for adults and older adults that were invariant across age; the only exception being that the reduction was statistically trending for older adults at end-of-treatment. Third and interestingly, statistically significant reductions in anxiety were observed at end-of-treatment and 12-months post-treatment but only for older adults. Fourth, for adults and older adults, high-impact chronic pain patients showed larger reductions in pain intensity and pain interference at end-of-treatment and 12-months post-treatment than lower impact chronic pain patients, although the high-impact chronic pain advantage was only statistically significant for adults due to restricted sample size in the older adult sample. At end-of-treatment, 81% of older adults and 68% of adult high-impact chronic pain patients were reclassified as lower impact chronic pain with 53% and 69% remaining reclassified as lower impact chronic pain at 12-months post-treatment. The high-impact chronic pain advantage also held for sleep and physical disability at both time points and for both age groups. The results were mixed for anxiety and depression.

The Aim 2 analysis yielded several interesting findings as well. VR program engagement in the RCT was higher for older adults than for the adult study sample (7). Notably, however, the older adult sample size was relatively small (*N* = 87) and thus gives rise to the question of selection bias. In other words, perhaps only the most motivated older adults joined our VR treatment study, and thus the results from our RCT would not generalize to other older adult populations. However, results from our clinical sample (*N* = 284) of older adults provides generalizable data with older adults evidencing high engagement with the clinically prescribed treatment that was larger than that observed for adults, thus replicating the results from the RCT. Average sessions completed ranged from 34.3 (of 56 total sessions in the therapy) for 18–64 adults in the real-world clinical sample to 46.4 for 65+ adults in the RCT. The engagement and adherence rates were high in both the real-world clinical and RCT samples as well. The engagement rate ranged from 63% for 18–64 adults in the clinical sample to 94% for 65+ adults in the RCT. Recall that the engagement rate denotes the percentage of patients who complete at least 24 sessions, which has been shown to lead to clinically meaningful pain reductions. A similar pattern held for the more stringent adherence rate with values ranging from 50% for 18–64 adults in the clinical sample to 85% for 65+ adults in the RCT. Importantly, regardless of whether the focus is on RCT or real-world clinical sample it was always the case that VR program engagement was higher for 65+ than 18–64 adults. As such, while acknowledging the potential selection bias in the randomized controlled trial sample we show that our real-world clinical sample of patients show high rates of older adult engagement with home-based VR and corresponding efficacy.

Although speculative, we offer three possible mechanisms to explain the older adult engagement advantage. First, the VR device likely has greater novelty for older adults than other adults. Research suggests that older adults show higher states of curiosity/interest (a proxy for perceived novelty) than adults toward the same new technology (33). Second, older adults may have more free time in their day to engage with the therapy (34). Finally, some studies suggest older adults are more compliant with medical instructions (35). Any one or a combination of these factors could explain the older adult engagement advantage.

Taken together, the results from the current study suggest that age did not affect treatment effectiveness at end-of-treatment or at 12-months post-treatment for this Skills-Based VR Therapy with both age groups obtaining meaningful reductions in pain intensity and pain interference that were robust to 12-months post-treatment. This age invariance held for most of the secondary endpoints at end-of-treatment and 12-months post-treatment and the clinical effectiveness advantage for high-impact chronic pain patients relative to lower impact chronic pain patients was robust for both age groups. Both the real-world clinical sample and the research sample suggest that age does not negatively impact patient engagement in the lab or in the wild and if anything, older adults show stronger VR program engagement. It is important to note that critical factors identified to mitigate potential age-based inequities are broad availability (e.g., no Wi-Fi necessary for the digital therapy to be delivered) and ease of use (e.g., minimal time commitment, in-home administration, and simple navigation) (6). The Skills-Based VR-Delivered Therapy incorporates these critical factors thus offering a powerful tool for mitigating chronic lower back pain that is self-administered in-home, requires no Wi-Fi connectivity to deliver the therapy, uses gaze-based navigation, and requires on average 6 min/day. It is important to note that broad availability and ease of use are not characteristics of all digital therapeutic programs, and if not present could attenuate engagement and subsequent clinical effectiveness in older adults and for other sociodemographic factors (e.g., gender, race/ethnicity, and socioeconomic status) (refs). Thus, it is critically important when developing and vetting digital therapeutics to evaluate availability and ease of use before recommending a specific digital therapeutic to patients (5–8).

### Limitations

Limitations to consider when evaluating the study results include the following: First, participants cannot be randomized to age groups and thus the results are correlational in nature. Second, the older adult sample size, *N* = 87, is modest making strong inference difficult. This limitation is mitigated somewhat by the inclusion of the large real-world clinical sample of older adults. Third, other potentially important factors not included in the analysis could be causal, such as pain-related and non-pain-related co-morbidities, among other factors. These should be the focus of future research. Fourth, self-reported chronic lower back pain was not confirmed by healthcare professionals. Finally, our study was specific to chronic lower back pain and results may not generalize to other populations.

## Conclusion

A pain relief skills VR program was associated with clinically meaningful benefits to up to one-year post-treatment, that were invariant across the adult and older adult age groups. VR program engagement in a 2,400+ real-world clinical sample was high and comparable to that observed in a large sample RCT. Across the real-world and RCT samples, VR program engagement was higher in older adults than adults. While further research is needed to understand how to best meet the needs of patients with CLBP, our results suggest that the home-based VR program tested here may be a scalable and effective treatment option.

## Data Availability

The raw data supporting the conclusions of this article will be made available by the authors, without undue reservation.

## References

[1] Ferreira ML de Luca K Haile LM Steinmetz JD Culbreth GT Cross M Global, regional, and national burden of low back pain, 1990–2020, its attributable risk factors, and projections to 2050: a systematic analysis of the global burden of disease study 2021. Lancet Rheumatol. (2023) 5:e316–29. 10.1016/S2665-9913(23)00098-X 37273833 PMC10234592

[2] Hansen LA Hartvigsen J Jensen RK. Differences in demographics and clinical outcomes in older, middle-aged, and younger adults with low back pain receiving chiropractic care. Chiropr Man Therap. (2025) 33:31. 10.1186/s12998-025-00589-w 40745315 PMC12315371

[3] Dahlhamer J Lucas J Zelaya, C Nahin R Mackey S DeBar L Prevalence of chronic pain and high-impact chronic pain among adults — united States, 2016. MMWR Morb Mortal Wkly Rep. (2018) 67:001–1006. 10.15585/mmwr.mm6736a2 PMC6146950 30212442

[4] Ames H Hestevik CH Briggs AM. Acceptability, values, and preferences of older people for chronic low back pain management; a qualitative evidence synthesis. BMC Geriatr. (2024) 24:1–22. 10.1186/s12877-023-04608-4 38182977 PMC10768085

[5] Seifert A Schlomann A. The use of virtual and augmented reality by older adults: potentials and challenges. Front Virtual Real. (2021) 2:1–5. 10.3389/frvir.2021.639718

[6] Seifert A Reinwand DA Schlomann A. Designing and using digital mental health interventions for older adults: being aware of digital inequality. Front Psychiatry. (2019) 10:1–4. 10.3389/fpsyt.2019.00568 31447716 PMC6696744

[7] Yao R Zhang W Evans R Cao G Rui T Shen L. Inequities in health care services caused by the adoption of digital health technologies: scoping review. J Med Internet Res. (2022) 24:1–16. 10.2196/34144 PMC8981004 35311682

[8] Mistry SK Shaw M Raffan F Johnson G Perren K Shoko S Inequity in access and delivery of virtual care interventions: a scoping review. Int J Environ Res Public Health. (2022) 19:9411. 10.3390/ijerph19159411 35954768 PMC9367842

[9] Maddox T Oldstone L Sackman J Judge E Maddox R Adair T Sociodemographic predictors of clinical effectiveness, therapeutic program engagement, and device usability for an in-home virtual reality program for chronic low back pain: secondary analysis of a randomized controlled trial. J Med Extend Reality. (2024) 1:65–72. 10.1089/jmxr.2023.0013 PMC13387221 42558092

[10] Maddox T Oldstone L Sparks CY Sackman J Oyao A Garcia L In-home virtual reality program for chronic lower back pain: a randomized sham-controlled effectiveness trial in a clinically severe and diverse sample. Mayo Clin Proc Digit Health. (2023) 1:563–73. 10.1016/j.mcpdig.2023.09.003 40206316 PMC11975703

[11] Silva S Pinto RZ Mendes G Santos RL Grade I de Mello MT Association between objective sleep and clinical outcomes in older adults with low back pain receiving physical therapy care: a secondary analysis of a responsiveness study. J Aging Phys Act. (2025) 33:251–61. 10.1123/japa.2024-0038 39566491

[12] Seo J Kim N Suk K-S Lee BH Bae Y Park M Increased risk of depression and anxiety in patients with chronic back pain following COVID-19 infection based on a nationwide population-based study. Sci Rep. (2025) 15:13333. 10.1038/s41598-025-95289-z 40246876 PMC12006292

[13] Tsatsaraki E Bouloukaki I Kontakis G Vakis AF Basta M. Associations between combined psychological and lifestyle factors with pain intensity and/or disability in patients with chronic low back pain: a cross-sectional study. Healthcare. (2023) 11:2928. 10.3390/healthcare11222928 37998420 PMC10671559

[14] Şentürk İA Şentürk E Üstün I Gökçedağ A Yıldırım NP İçen NK. High-impact chronic pain: evaluation of risk factors and predictors. Korean J Pain. (2023) 36:84–97. 10.3344/kjp.22357 36581599 PMC9812691

[15] Pitcher MH Von Korff M Bushnell MC Porter L. Prevalence and profile of high-impact chronic pain in the United States. J Pain. (2019) 20:146–60. 10.1016/j.jpain.2018.07.006 30096445 PMC8822465

[16] Vaegter HBP Due Bruun KMP Bye-Møller LP. High-impact chronic pain. Int Assoc Study Pain. (2023).

[17] Maddox T Oldstone L Linde-Zwirble W Bonakdar R Maddox R Sackman J Differential treatment response to virtual reality in high-impact chronic pain: secondary analysis of a randomized trial. Sci Rep. (2025) 15:14430. 10.1038/s41598-025-98716-3 40281004 PMC12032132

[18] Lucas JW Sohi I. Chronic pain and high-impact chronic pain among U.S. adults, 2023. NCHS Data Brief. (2024):CS355235. 10.15620/cdc/169630 39751180 PMC11726267

[19] Maddox T Oldstone L Sackman J Maddox R Adair T Ffrench K 12-month results for a randomized sham-controlled effectiveness trial of an in-home skills-based virtual reality program for chronic low back pain. Pain Rep. (2024) 9:e1182. 10.1097/PR9.0000000000001182 39239633 PMC11377093

[20] Cleeland CS Ryan KM. Pain assessment: global use of the brief pain inventory. Ann Acad Med Singap. (1994) 23:129–38. 8080219

[21] Birckhead B Khalil C Liu X Conovitz S Rizzo A Danovitch I Recommendations for methodology of virtual reality clinical trials in health care by an international working group: iterative study. JMIR Ment Health. (2019) 6:e11973. 10.2196/11973 30702436 PMC6374734

[22] Cella D Yount S Rothrock N Gershon R Cook K Reeve B The patient-reported outcomes measurement information system (PROMIS): progress of an NIH roadmap cooperative group during its first two years. Med Care. (2007) 45:S3–S11. 10.1097/01.mlr.0000258615.42478.55 17443116 PMC2829758

[23] Yu L Buysse DJ Germain A Moul DE Stover A Dodds NE Development of short forms from the PROMIS™ sleep disturbance and sleep-related impairment item banks. Behav Sleep Med. (2011) 10:6–24. 10.1080/15402002.2012.63626 22250775 PMC3261577

[24] Fairbank JCT Pynsent PB. The oswestry disability Index. Spine. (2000) 25:2940–53. 10.1097/00007632-200011150-00017 11074683

[25] Laird NM Ware JH. Random-effects models for longitudinal data. Biometrics. (1982) 38:963. 10.2307/2529876 7168798

[26] Cnaan A Laird NM Slasor P. Using the general linear mixed model to analyse unbalanced repeated measures and longitudinal data. Stat Med. (1997) 16:2349–80. 10.1002/(sici)1097-0258(19971030)16:20<2349::aid-sim667>3.0.co;2-e 9351170

[27] Cook KF Dokyoung SY Von Korff M Mackey SC. Defining chronic pain impact levels: a patient-clinician approach using PROMIS® pain interference scores. J Patient Reported Outcomes. (2025) 9:1–11. 10.1186/s41687-025-00908-y PMC12361012 40824460

[28] Garcia LM Birckhead BJ Krishnamurthy P Sackman J Mackey IG Louis RG An 8-week self-administered at-home behavioral skills-based virtual reality program for chronic low back pain: double-blind, randomized, placebo-controlled trial conducted during COVID-19. J Med Internet Res. (2021) 23:e26292. 10.2196/26292 33484240 PMC7939946

[29] Maddox T Sackman J Stoudt M Chibbaro M Judge E Rothery R Virtual reality-delivered skills-based therapy for chronic lower back pain: from proof-of-concept to FDA authorization to clinical implementation in-home and beyond. Front Virtual Real. (2025) 6:1–20. 10.3389/frvir.2025.1613188

[30] Saleem M Kühne L De Santis KK Christianson L Brand T Busse H. Understanding engagement strategies in digital interventions for mental health promotion: scoping review. JMIR Ment Health. (2021) 8:e30000. 10.2196/30000 34931995 PMC8726056

[31] Graham AK Kwasny MJ Lattie EG Greene CJ Gupta NV Reddy M Targeting subjective engagement in experimental therapeutics for digital mental health interventions. Internet Interv. (2021) 25:100403. 10.1016/j.invent.2021.100403 34401363 PMC8350581

[32] Elkes J Cro S Batchelor R O’Connor S Yu L-M Bell L User engagement in clinical trials of digital mental health interventions: a systematic review. BMC Med Res Methodol. (2024) 24:184. 10.1186/s12874-024-02308-0 39182064 PMC11344322

[33] Chu L Fung HH. Age differences in state curiosity: examining the role of personal relevance. Gerontology. (2022) 68:321–9. 10.1159/000516296 34062532

[34] Marcum CS. Age differences in daily social activities. Res Aging. (2013) 35:612–40. 10.1177/0164027512453468 25190898 PMC4151480

[35] Ge L Heng BH Yap CW. Understanding reasons and determinants of medication non-adherence in community-dwelling adults: a cross-sectional study comparing young and older age groups. BMC Health Serv Res. (2023) 23:1–11. 10.1186/s12913-023-09904-8 37620970 PMC10464472

